# Close-to-lesion transbronchial biopsy: a novel technique to improve suitability of specimens for genetic testing in patients with peripheral pulmonary lesions

**DOI:** 10.1038/s41598-023-41726-w

**Published:** 2023-09-07

**Authors:** Yoichi Nishii, Tadashi Sakaguchi, Seiya Esumi, Maki Esumi, Yuki Nakamura, Yuta Suzuki, Kentaro Ito, Kentaro Fujiwara, Hiroki Yasui, Atsushi Ito, Tomohito Tarukawa, Tatsuki Tsuruga, Corina N. D’Alessandro-Gabazza, Taro Yasuma, Hajime Fujimoto, Fumihiro Asano, Esteban C. Gabazza, Tetsu Kobayashi, Osamu Taguchi, Osamu Hataji

**Affiliations:** 1 Respiratory Center, Matsusaka Municipal Hospital, Tonomachi 1550, Matsusaka, Mie 515-8544 Japan; 2 Department of Pulmonary and Critical Care Medicine, Mie University Faculty and Graduate School of Medicine, Edobashi 2-174, Tsu, Mie 514-8507 Japan; 3 Department of Immunology, Mie University Faculty and Graduate School of Medicine, Edobashi 2-174, Tsu, Mie 514-8507 Japan; 4 Gifu Prefectural General Medical Center, Noisshiki 4-6-1, Gifu, Gifu 500-8717 Japan

**Keywords:** Cancer, Diseases, Oncology

## Abstract

Bronchoscopy with radial-probe endobronchial ultrasound, a guide sheath, and electromagnetic navigation can improve the diagnostic yield of peripheral lung nodules. However, the suitability of specimens for genetic analysis remains unsatisfactory. We hypothesized that a transbronchial biopsy performed after closely approaching the bronchoscope tip to the lesion might provide more suitable specimens for genetic analysis. We enrolled 155 patients with peripheral pulmonary lesions who underwent bronchoscopy with a thin or ultrathin bronchoscope. Bronchoscopy was performed using virtual bronchoscopic navigation and radial-probe endobronchial ultrasound with a guide sheath. The bronchoscope tip was placed closer to the lesion during bronchoscopy to collect larger specimens with higher malignant cell content. The patients who underwent a close-to-lesion biopsy had higher rates of overall diagnostic yield, histopathological diagnostic yield, and specimen quality for genetic testing than those who did not. The significant determinants of the specimen’s suitability were the close-to-lesion approach, within-the-lesion image, the use of standard 1.9-mm-forceps, and the number of cancer-cell-positive specimens. The significant predictors of the specimen’s suitability for genetic analysis were close-to-lesion biopsy and the number of malignant cell-positive tissue samples. This study demonstrates that the close-to-lesion transbronchial biopsy significantly improves the suitability of bronchoscopic specimens for genetic analysis.

## Introduction

In recent years, the accuracy of computed tomography (CT) has led to increased detection of small peripheral pulmonary lesions. The diagnostic yield of pulmonary lesions less than 2 cm is low using conventional bronchoscopic methods. However, the introduction of virtual bronchoscopic navigation (VBN) and radial-probe endobronchial ultrasound (RP-EBUS) with guide sheath has significantly improved the diagnostic yield of peripheral pulmonary lesions^1–3^. The diagnostic yield of subsegmental pulmonary lesions ≤ 30 mm is 80.4% when VBN and RP-EBUS are used in combination^4^. The American College of Chest Physicians (ACCP) guideline recommends using RP-EBUS or electromagnetic navigation (EMN) because these techniques substantially improve the diagnostic yield^5^. The RP-EBUS image of the lesion during bronchoscopy has a diagnostic significance. The diagnostic yield is 83% when the image is within the lesion, 61% when adjacent to the lesion, and only 4% when outside the lesion^6^. Transbronchial biopsy performed with an RP-EBUS image within the lesion provides the highest diagnostic yield; however, there are cases where the diagnosis cannot be confirmed due to low quality and inappropriate volume of biopsied tissues. Improper tissue sampling is also a major obstacle to achieving a successful genetic analysis of lung cancer using next-generation sequencing (NGS). NGS has become a necessary test for expanding therapeutic options in patients with non-small cell lung cancer (NSCLC), so it is essential to ensure that an appropriate volume and high-quality tissue samples are collected during transbronchial biopsy^7^. Large 1.9-mm forceps and cryo-probes are recommended for this purpose^7,8^. However, tissue sampling of peripheral pulmonary lesions with 1.9-mm forceps and cryoprobes is generally not performed because bronchoscopes with small diameters are routinely used in clinical practice.

The suitability for NGS of specimens collected by bronchoscopy in lung cancer patients with peripheral pulmonary lesions is unclear. Previous studies have reported the outcomes of NGS analysis using tissue samples obtained by combining different methods, including transbronchial biopsy, computed tomography-guided needle biopsy, or surgical resection in patients with lung cancer. However, there is no data on the suitability and success of NGS analysis using tissue samples collected by bronchoscopy in lung cancer patients with peripheral pulmonary lesions. The volume and the malignant cell content of the collected specimen are crucial for the success of NGS analysis^7^. We hypothesize that bringing the bronchoscope tip closer to the lesion will allow us to collect larger specimens with higher malignant cell content and improve the suitability for genetic testing of specimens collected from non-small cell lung cancer (NSCLC) patients with peripheral pulmonary lesions. To test this hypothesis, we propose a modified bronchoscopic technique that differs from the conventional procedure as follows: after confirming the lesion position with the RP-EBUS, we use the guide sheath as a rail to insert the bronchoscope tip closer to the lesion under RP-EBUS and fluoroscopy guidance. Then, we remove the RP-EBUS and the guide sheath after re-confirming the lesion position and introduce a 1.9-mm forceps for tissue sampling. Using this novel approach, we assessed the diagnostic yield of peripheral pulmonary lesions and the suitability rate for NGS analysis of the collected tissue samples.

## Materials and methods

### Subjects

**Figure 1:**
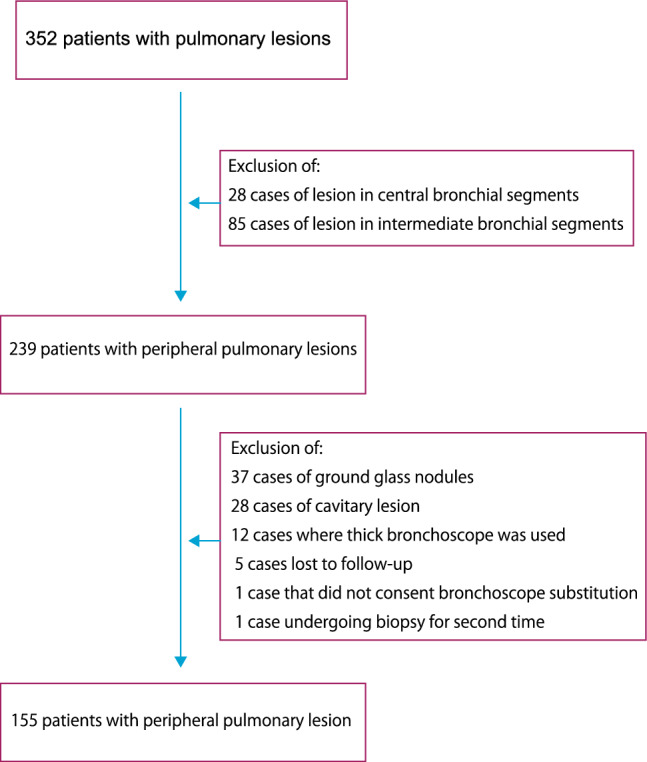
Study selection criteria. The clinical records of 352 patients that underwent bronchoscopy for parenchymal lesions were evaluated. Of these, 155 were eligible for the study after excluding patients with central or intermediate lung segmental lesions, ground glass nodules, cavitary lesions, patients undergoing bronchoscopy using thick bronchoscopes, and those lost to follow-up.

This study enrolled 155 patients with peripheral pulmonary lesions that underwent bronchoscopy using a thin bronchoscope with RP-EBUS and guide sheath or an ultrathin bronchoscope with RP-EBUS from September 2021 through November 2022 (Fig. 1). The Ethics Committee for Clinical Investigation of Matsusaka Municipal Hospital approved the study protocol (Approval No.: j-241-230519-1-1), and the study was performed following the Principles of the Helsinki Declaration. Written informed consent was obtained from all study participants.

### Study design

The current work is a retrospective and a single-institution study. The following variables were evaluated: patients’ gender and age, computed tomography (CT) bronchus sign, overall and histopathological diagnoses, bronchoscope tip close-to-lesion biopsy, and lesion size, RP-EBUS image, chest X-ray finding, fluoroscopy visualization, and lung distribution (2.5 cm from the hilum/intermediate/2.5 cm from the pleura)^9^.

### Materials

All patients underwent CT (TSX-101A/QA, Canon Medical Corporation, Tochigi, Otawara, Japan) prior to surgery, and VBN images were generated using CT-Digital Imaging and Communications in Medicine (CT-DICOM) data (DirectPath; Cybernet Systems, Tokyo, Japan). Bronchoscopy was performed using the thin flexible bronchoscope type P290 (outer diameter of 4.2 mm; working channel diameter of 2.0 mm; Olympus Medical Systems, Tokyo, Japan) or the ultrathin bronchoscope type MP290F (outer diameter of 3.0 mm; working channel diameter of 1.7 mm; Olympus Medical Systems, Tokyo, Japan). Topical lidocaine was used for pharyngeal anesthesia, while intravenous midazolam and fentanyl were administered for patient sedation. The airways were nebulized with lidocaine using a 1.9-mm spray catheter (Spray Catheter; Olympus, Tokyo, Japan), and additional intravenous fentanyl was administered as needed. Bronchoscopy with a thin or ultrathin bronchoscope was conducted with VBN, fluoroscopy, and 1.4-mm RP-EBUS (UM-S2-17S, Olympus, Tokyo, Japan) with or without a 1.95-mm guide sheath (SG-200C, Olympus, Tokyo, Japan). Biopsy was performed using a 1.5-mm (FB-233D; Olympus, Tokyo, Japan) or a 1.9-mm forceps (FB-231D, Olympus, Tokyo, Japan). Bronchial brushing and washing with 20 mL of physiological saline were performed after a close-to-lesion transbronchial biopsy (CL-TBB). As previously described, RP-EBUS images were categorized as within or adjacent to the lesion or invisible^3^.

### Transbronchial biopsy and NGS analysis

**Figure 2:**
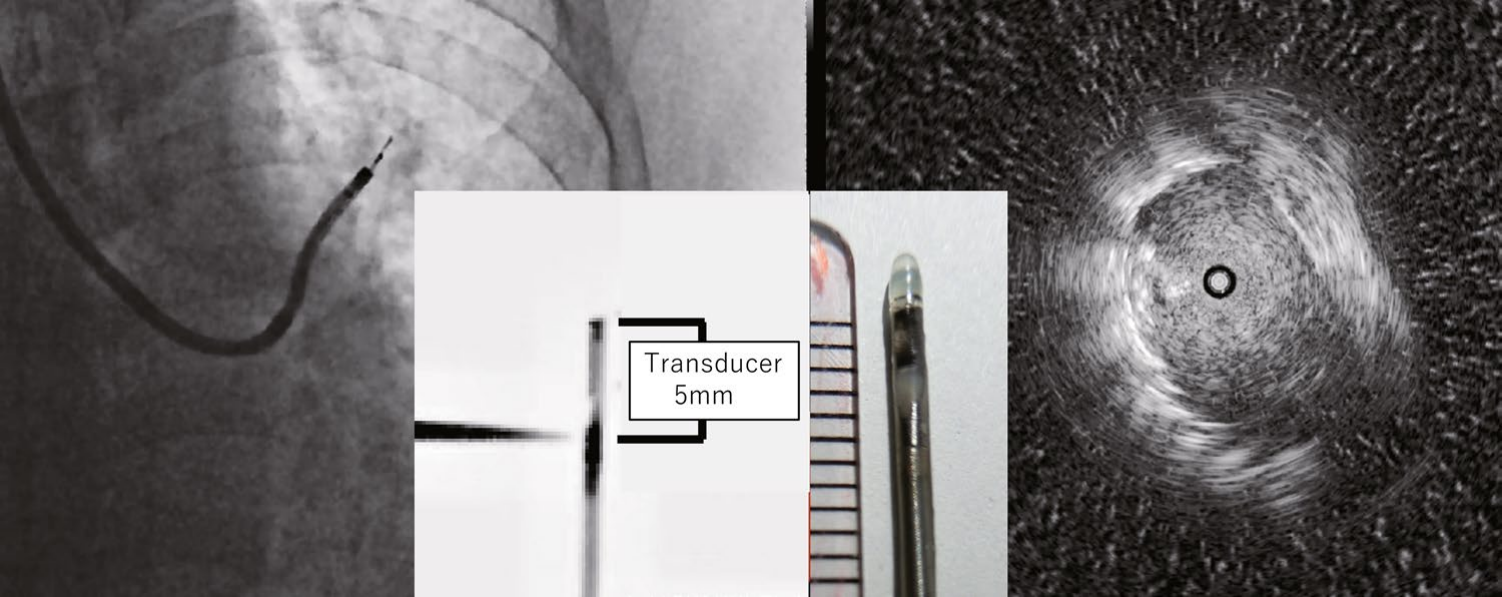
Criteria for CL-TBB. The distance from the RP-EBUS probe tip to the ultrasound transducer is 5 mm. “CL-TBB” was considered when the distance from the bronchoscope tip to the lesion was ≤ 10 mm under fluoroscopy and RP-EBUS images.

A tissue sampling was defined as a CL-TBB when the bronchoscope tip was within or adjacent to the lesion by RP-EBUS and ≤ 10 mm from the lesion under fluoroscopy (Fig. 2). We assessed the NGS suitability rate, the NGS analysis success rate, and the overall diagnostic yield of the technique. The NGS suitability rate was the proportion of samples that met the criteria for genetic analysis, and the NGS analysis success rate was the proportion of samples that allowed both DNA and RNA analysis. We fixed the biopsy specimens immediately with 10% neutral-buffered formalin at room temperature for 12–24 h and then embedded them in paraffin. An experienced pathologist evaluated the number of tumor cells in specimens stained with hematoxylin and eosin. The sample was suitable for genetic analysis when the number of tumor cells in the specimen was ≥ 200 or the proportion of tumor cells in the specimen was ≥ 30%. The attending physician judged the suitability of the sample for NGS analysis based on the clinical findings when the number of tumor cells was between 100 and 200. We sent the samples to LSI Medience Laboratories (Tokyo, Japan) to perform NGS analysis using Oncomine Dx Target Test (ODxTT; Ion Torrent PGM Dx Sequencer; Thermo Fisher Scientific)^10^.

### Diagnosis

The final diagnosis in all cases was made based on histopathological, cytological, microbiological, culture, and clinical findings. Samples collected by bronchoscopy (lung tissue, bronchial washing, or brushing), CT-guided lung biopsy, or surgery were used for pathological diagnosis. Lung cancer was diagnosed when any of these samples confirmed malignancy. Organizing pneumonia was diagnosed when the bronchoscopy showed no significant findings, CT-guided lung biopsy or surgery was not performed, or a subsequent CT study confirmed the disappearance or shrinkage of the lesion.

### CL-TBB using a thin bronchoscope

**Figure 3:**
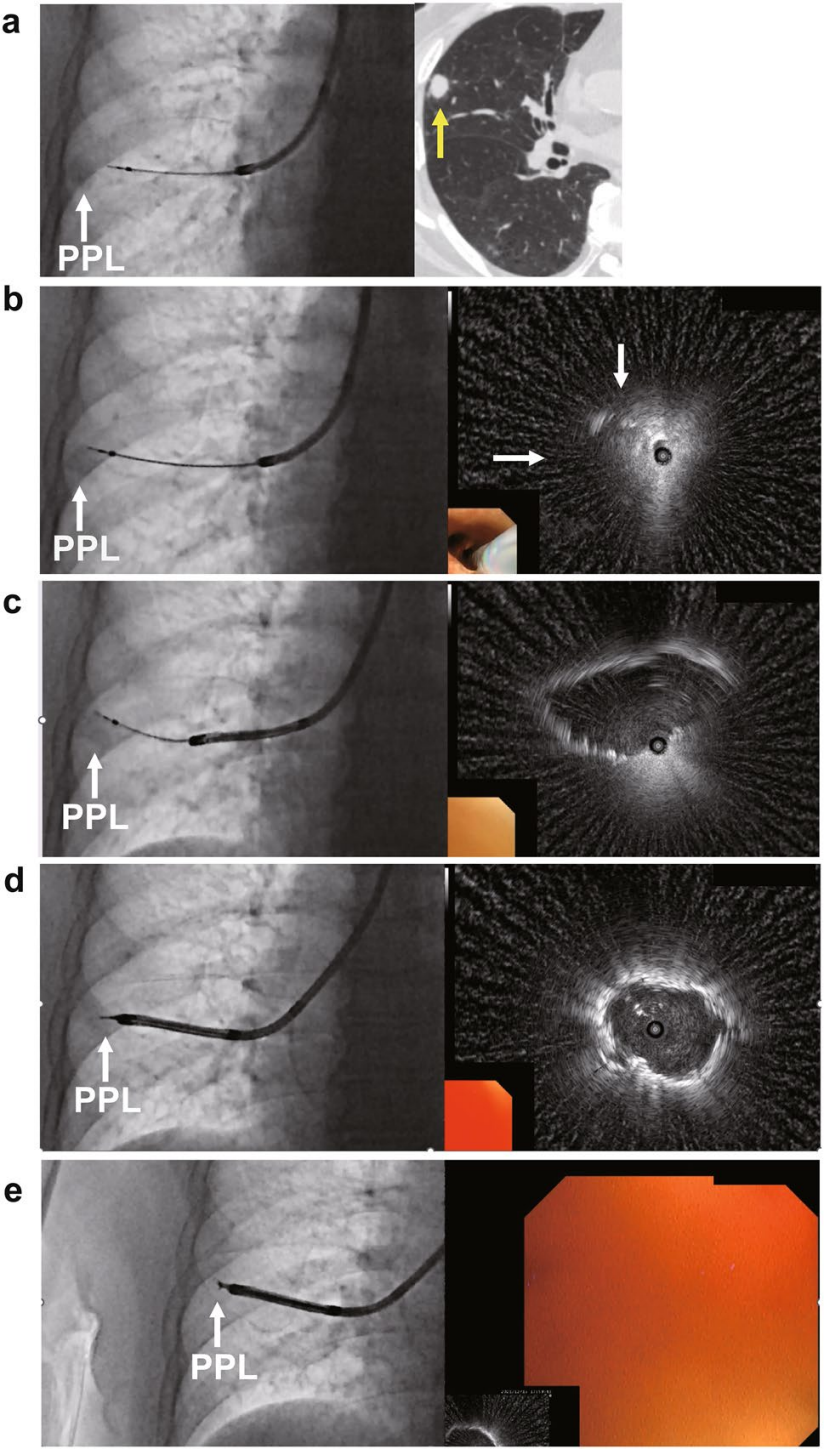
CL-TBB using a thin bronchoscope. (**a**) A patient with a peripheral pulmonary lesion. (**b**) The bronchoscope is inserted, and the RP-EBUS image shows that the probe is adjacent to the lesion. (**c**) The bronchoscope tip is moved closer to the lesion under RP-EBUS and fluoroscopy guidance. (**d**) The bronchoscope tip is pushed further using the RP-EBUS-guide sheath as a rail while checking the RP-EBUS image for the lesion position. The bronchoscope is moved in different directions to get the RP-EBUS image within the lesion. (**e**) The RP-EBUS is removed, and a 1.5-forceps is inserted through the guide sheath to collect 5 tissue samples. The guide sheath is removed, and a standard 1.9-mm forceps is inserted to collect 5 more tissue samples.

The thin bronchoscope was advanced through the bronchus segment and positioned as close to the lesion as possible under the airway lumen and VBN image guidance. Then, the RP-EBUS with the guide sheath was inserted through the 2-mm working channel of the thin bronchoscope until the RP-EBUS-guide sheath tip reached the lesion, and this can be visualized and confirmed by RP-EBUS. When the RP-EBUS image showed within or adjacent to the lesion, the bronchoscope tip was further advanced as close to the lesion as possible using the RP-EBUS-guide sheath as a rail under RP-EBUS and fluoroscopy guidance (Fig. 3, Supplementary Video). During this close approach to the lesion, an assistant holds the RP-EBUS-guide sheath while the bronchoscopist further advances the bronchoscope tip toward the lesion of interest using the RP-EBUS-guide sheath as a rail. After confirming that the bronchoscope tip was close to the lesion and within or adjacent to the lesion by RP-EBUS image, the RP-EBUS was removed, leaving the guide sheath in position. A 1.5-mm forceps was then inserted through the guide sheath, following the same route toward the lesion to collect five pieces of the tissue sample. After collecting the samples and confirming that the lesion was visualized by fluoroscopy and the RP-EBUS image was within or adjacent to the lesion, the guide sheath was removed to introduce a 1.9-mm forceps and collect five additional biopsy samples. Therefore, a total of 10 visible tissue samples were collected.

### CL-TBB using an ultrathin bronchoscope

The thin bronchoscope was substituted by the ultrathin bronchoscope when the close-to-lesion approach was impossible because the lesion was invisible by RP-EBUS or because the area of contact with the lesion was less than 180° when the RP-EBUS image was adjacent to the lesion. Neither the guide sheath (1.95-mm) nor the standard (1.9-mm) forceps can be used with the ultrathin flexible bronchoscope because its working channel is only 1.7-mm in diameter. Therefore, the biopsy was performed after bronchoscope substitution using the 1.5-mm forceps under RP-EBUS (1.4-mm) and fluoroscopy guidance. The technique for approaching close to the lesion was similar to that used with the thin bronchoscope. The ultrathin bronchoscope was advanced through the bronchial segments toward the lesion under RP-EBUS guidance until the bronchoscope tip was as close to the lesion as possible. The RP-EBUS was then inserted through the bronchoscope working channel until the RP-EBUS tip reached the lesion area, and the lesion could be visualized and confirmed by fluoroscopy and RP-EBUS. After confirming the RP-EBUS image was within or adjacent to the lesion, the RP-EBUS was withdrawn to introduce a 1.5-mm forceps and collect ten tissue samples.

### Evaluation and prevention of complications

Medical records were examined for procedure-related complications within one month after the bronchoscopy. After the transbronchial biopsy, the forceps was withdrawn, but the bronchoscope tip or guide sheath was kept securely in the bronchial branch for 3 min for compressive hemostasis to prevent bleeding.

### Statistical analysis

SPSS Statistics version 23 software (SPSS Inc., Chicago, IL, USA) was used for statistical analysis. The chi-square test was used to compare proportions between groups, and the Mann–Whitney U test was used to analyze continuous variables. Logistic regression analysis was used to identify factors that can predict the suitability of collected specimens for NGS analysis. A two-sided *P* < 0.05 was considered significant.

## Results

### There were 155 eligible patients

During the investigation period, 352 patients underwent bronchoscopy for lung parenchymal lesions. However, only 155 were eligible for the study after excluding patients with central (n = 28) or intermediate (n = 85) lung segmental lesions, ground glass nodules (n = 37), cavitary lesions (n = 28), or patients undergoing examination with thick bronchoscope (n = 12). We also excluded a case undergoing a second biopsy, a patient who did not consent to substitute the bronchoscope, and those lost to follow-up (n = 5) (Fig. 1).

**Figure 4:**
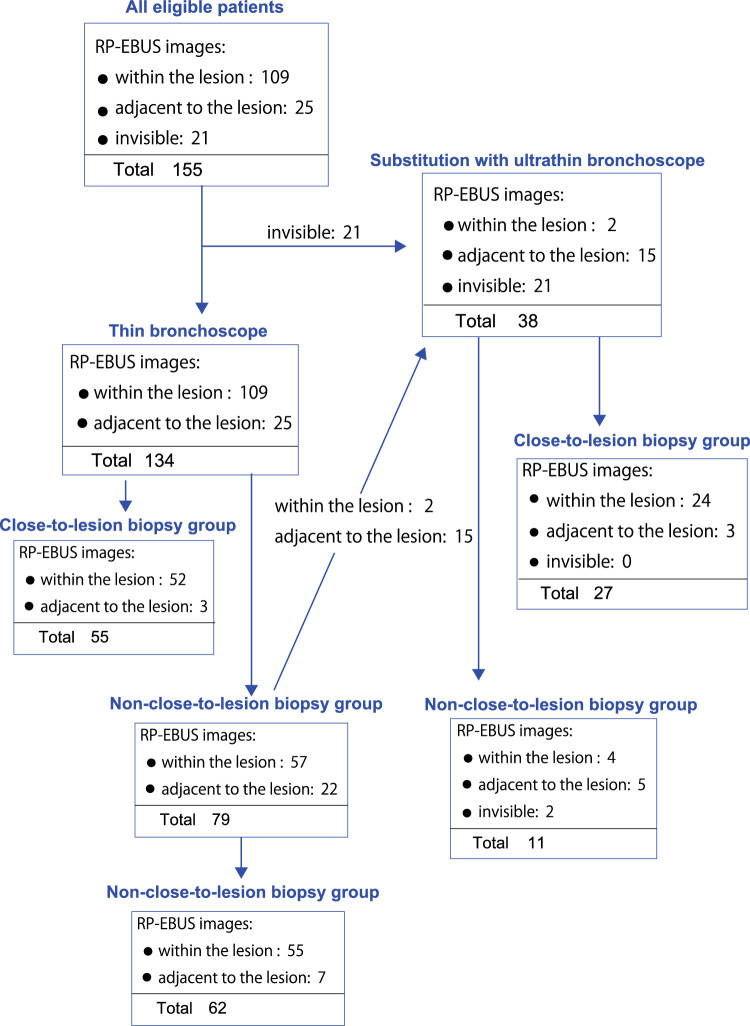
Eligible patients for the study. A total of 155 patients were included in the study. Of these, 134 patients had radial probe endobronchial ultrasound (RP-EBUS) images showing “within-the-lesion” or “adjacent-to-the-lesion” and underwent bronchoscopy using a thin bronchoscope. The remaining 21 patients had lesions that were invisible by RP-EBUS and underwent bronchoscopy using an ultrathin bronchoscope. CL-TBB was possible in 55 of the 134 patients who underwent bronchoscopy with a thin bronchoscope. The thin bronchoscope was replaced with the ultrathin bronchoscope in 17 patients who could not undergo CL-TBB. CL-TBB was possible in 27 patients who underwent bronchoscopy with an ultrathin bronchoscope (RP-EBUS images showing invisible lesions in 21 cases, within the lesion in 2, and adjacent to the lesion in 15 cases).

The median size of peripheral pulmonary lesions in the eligible patients was 19 (5 ~ 60) mm. Among all 155 patients, 81.3% had lesions confirmed by fluoroscopy, and 80.0% showed a CT bronchus sign. Thirty-eight (24.5%) required the substitution of the thin bronchoscope with an ultrathin bronchoscope because the lesion was invisible by RP-EBUS (n = 21), sample collection was not possible by forceps bending (n = 2) in patients with RP-EBUS image within the lesion, or the area of contact with the lesion was less than 180° in patients (n = 15) with RP-EBUS image adjacent to the lesion (Table 1, Fig. 4). The RP-EBUS image showed within the lesion in 87.1% of cases, adjacent in 11.6%, and invisible in 1.3% of cases. The overall high visual yield (98.7%, within lesion + adjacent to the lesion) may be attributed to the active substitution of the thin bronchoscope with an ultrathin bronchoscope and the close-to-lesion approach.

**Table 1:** Characteristics of 155 eligible patients.

Variables	No. of cases/data	Percentage of cases
Age (median and range)	74 (29 ~ 92)	
Male/female	109/46	
Lesion size mm (median and range)	19 (5 ~ 60)	
< 10 mm	8	5.2
> 10 mm < 20 mm	78	50.3
> 20 mm	69	44.5
Detectable by chest X-ray	106	68.4
Visible by fluoroscopy	126	81.3
CT bronchus sign (+)	124	80.0
Virtual bronchoscopic navigation	155	100
Lesion location		
Right upper lobe	44	28.4
Right middle lobe	16	10.3
Right lower lobe	40	25.7
Left upper lobe	39	24.2
Left lower lobe	16	10.3
Substitution with an ultrathin bronchoscope	38	24.5
Use of standard 1.9-mm forceps (thin bronchoscope)	78	50.3
Final diagnosis		
Malignant disease	108	69.7
Lung cancer	100	64.5
Adenocarcinoma	60	38.9
SCC	23	14.8
Undefined NSCLC	11	7.1
SCLC	6	3.9
Lung metastasis	6	3.9
Colon carcinoma	3	1.9
Gastric carcinoma	1	0.6
Renal cell carcinoma	1	0.6
Pancreatic carcinoma	1	0.6
Malignant lymphoma	2	1.3
Benign disease	39	25.2
Organizing pneumonia	20	12.9
Nontuberculous Mycobacteriosis	13	8.4
Tuberculosis	3	1.9
Lung fluke disease	1	0.6
Pulmonary embolism	1	0.6
Eosinophilic pneumonitis	1	0.6
Not defined	8	5.2
RP-EBUS images		
Within the lesion	135	87.1
Adjacent to the lesion	18	11.6
Invisible	2	1.3
Overall diagnostic yield	128	82.6
Histopathological diagnostic yield	115	74.2

*SCC* squamous cell carcinoma, *SCLC* small cell lung cancer, *NSCLC* non-small cell lung cancer.

The overall diagnostic yield was 82.6%, and the histopathological diagnostic yield was 74.2%. There were 108 (69.7%) patients diagnosed with a malignant lung tumor (NSCLC n = 94, SCLC n = 6, metastatic lung tumor n = 6, malignant lymphoma n = 2), and 39 (25.2%) with benign lung disease. A final diagnosis was not achieved in 8 cases (Table 1). The diagnosis of malignant tumors or benign diseases such as infections by cytology or culture study of samples other than lung tissue (e.g., bronchial lavage fluid) may explain the difference between the overall and histopathological diagnostic yields.

### CL-TBB improved histopathological diagnostic yield

Close-to-lesion biopsy was possible in 82 patients (52.9%), as shown in Fig. 4 and Table 2. The pulmonary lesion was more significantly visible under fluoroscopy in patients that underwent CL-TBB than those who did not. Moreover, the overall diagnostic yield was better, and the histopathological diagnostic yield was significantly higher (81.7 vs. 65.8%, *P* = 0.023) in the CL-TBB group than in the non-CL-TBB group. However, the lesion size was not significantly different between both groups (Table 2).

**Table 2:** Comparison between CL-TBB and non-CL-TBB groups in all eligible patients.

Variables	CL-TBB group	Non-CL-TBB group	*P* values
No of patients	82 (52.9%)	73 (47.1%)	
Age (median and range)	74.5 (29 ~ 92)	74 (43 ~ 87)	0.403
Male/female	59/23	50 / 23	0.638
Lesion size mm (median and range)	20 (10 ~ 60)	18 (5 ~ 59)	0.816
< 10 mm	6 (7.3%)	2 (3.3%)	0.199
> 10– < 20 mm	38 (46.3%)	40 (56.8%)	0.293
> 20 mm	38 (46.3%)	31 (42.5%)	0.628
Location			
Right upper lobe	19 (23.3%)	26 (35.6%)	0.088
Right middle lobe	10 (12.2%)	6 (8.2%)	0.417
Right lower lobe	23 (28.0%)	17 (23.3%)	0.499
Left upper lobe	20 (24.4%)	18 (24.7%)	0.969
Left lower lobe	10 (12.2%)	6 (8.2%)	0.417
Both lower lobes	33 (40.2%)	23 (31.5%)	0.113
Lesion visible by chest X-ray	53 (64.6%)	53 (72.6%)	0.277
Lesion visible by fluoroscopy	80 (97.6%)	46 (63.0%)	0.001
CT bronchus sign (+)	65 (79.3%)	59 (80.8%)	0.809
RP-EBUS image within the lesion	76 (92.7%)	59 (80.8%)	0.028
Thin bronchoscope	55 (67.1%)	62 (84.9%)	0.01
Overall diagnostic yield	50 (90.9%)	50 (80.6%)	0.116
Histopathological diagnostic yield	46 (83.6%)	43 (69.4%)	0.071
Ultrathin bronchoscope	27 (32.9%)	11 (13.4%)	0.01
Overall diagnostic yield	22 (81.5%)	6 (54.5%)	0.087
Histopathological diagnostic yield	21 (77.8%)	5 (45.5%)	0.052
Overall diagnostic yield	72 (87.8%)	56 (76.7%)	0.069
Histopathological diagnostic yield	67 (81.7%)	48 (65.8%)	0.023

*CL-TBB* close-to-lesion transbronchial biopsy, *CT* computed tomography, *RP-EBUS* radial probe endobronchial ultrasound.

### CL-TBB improved the specimen's suitability

Among patients with NSCLC (n = 94), adenocarcinoma was more frequent than squamous cell carcinoma. Moreover, specimens collected from patients who underwent CL-TBB showed a significantly higher NGS suitability rate (71.4 vs. 47.4%, *P* = 0.019) than specimens collected from patients who did not undergo CL-TBB. In addition, the lesion visibility under fluoroscopy (98.2 vs. 68.4%, *P* < 0.0001) was also significantly increased in the CL-TBB group compared to the non-CL-TBB group (Table 3).

**Table 3:** Comparative evaluation between CL-TBB and non-CL-TBB groups in NSCLC patients.

Variables	CL-TBB group (n = 56)	Non-CL-TBB group (n = 38)	*P* value
Lesion size mm (median and range)	21.5 (6 ~ 50)	18 (10 ~ 40)	0.158
Lesion visible by chest X-ray	39 (69.6%)	27 (71.1%)	0.883
Lesion visible by fluoroscopy	55 (98.2%)	26 (68.4%)	0.001
CT bronchus sign (+)	47 (83.9%)	28 (73.7%)	0.225
RP-EBUS image within the lesion	52 (92.9%)	31 (81.6%)	0.095
Overall diagnostic yield	53 (94.6%)	33 (86.8%)	0.183
Histopathological diagnostic yield	49 (87.5%)	30 (78.9%)	0.266
Use of Standard 1.9-mm forceps	35 (62.5%)	19 (50.0%)	0.229
No of positive tissue median (range)	5 (0–10)	4 (0–10)	0.356
NGS suitability rate	40 (71.4%)	18 (47.4%)	0.019
NGS analysis success rate (in suitable samples)	40 (100.0%)	18 (100.0%)	1.00

*CL-TBB* close-to-lesion transbronchial biopsy, *RP-EBUS* radial probe endobronchial ultrasound, *NGS* next-generation sequencing.

However, no difference in lesion size or frequency of CT bronchus signs was observed between the CL-TBB and non-CL-TBB groups. In addition, the overall diagnostic yield and histopathological diagnostic yield, use of standard 1.9-mm forceps use, and the number of cancer cell-positive tissue samples were increased in patients that underwent CL-TBB compared to those who did not, although the difference was not statistically significant (Table 3).

### CL-TBB predicts the specimen's suitability

The importance of several variables for the suitability of specimens for NGS analysis was evaluated by univariate and multivariate analysis. The univariate analysis showed that CL-TBB, RP-EBUS image within the lesion, use of a standard 1.9-mm forceps, and the number of tissue samples with malignant cells were significant determinant factors for the suitability of collected specimens for NGS analysis (Table 4). However, the lesion size and CT bronchus sign were not significant factors for the NGS analysis suitability of the specimens. Then, we performed a logistic regression analysis, including variables that showed significant differences in the univariate analysis. CL-TBB and the number of samples with malignant cells were significant predictors of the suitability of collected specimens for NGS analysis (Table 4).

**Table 4:** Determinant and predictor factors of suitability of collected specimens for next-generation sequencing analysis in patients with non-small cell lung cancer.

	Suitable for NGS analysis	Unsuitable for NGS analysis	*P* values
Number of patients	58	36	
Univariate analysis			
CL-TBB	40 (69.1%)	16 (44.4%)	0.019
Lesion size mm, median (mean ± SD)	20.5 ± 10.3	18.0 ± 9.6	0.729
RP-EBUS image within the lesion	56 (96.6%)	27 (75.0%)	0.002
Lesion visible by chest X-ray	41 (70.7%)	25 (74.3%)	0.898
Lesion visible by fluoroscopy	51 (88.0%)	30 (83.3%)	0.530
CT bronchus sign	47 (81.0%)	28 (77.8%)	0.702
Standard 1.9-mm forceps	39 (67.2%)	15 (41.7%)	0.015
No of positive tissue (median)	6	1.5	0.0001

	Odds ratio		*P* values
Multivariate regression analysis			
CL-TBB	4.764		0.032
RP-EBUS image within the lesion	2.050		0.469
Standard 1.9-mm forceps	1.765		0.387
Number of samples with malignant cells	2.198		0.001

*CL-TBB* close-to-lesion transbronchial biopsy, *NGS* next-generation sequencing, *RP-EBUS* radial probe endobronchial ultrasound, *CT* computed tomography.

### NGS analysis success rate

The NGS analysis success rate of suitable specimens from 58 patients was 100% (Table 3). This indicates that CL-TBB is effective for obtaining high-quality specimens for genetic testing.

Collection of specimens for NGS analysis by bronchoscopy was not possible in 8 patients. Of these, 3 patients had alternative methods for specimen collection: CT-guided needle biopsy in 2 and surgery for stage IIIA lung cancer in 1 patient. The remaining 5 cases did not consent further procedures for specimen collection.

### Reduced complications after close-to-lesion biopsy

Complications were graded according to the Common Terminology Criteria for Adverse Events (CTCAE v5; accessed on August 8, 2023) https://ctep.cancer.gov/protocolDevelopment/electronic_applications/ctc.htm#ctc_50). Only two patients in the CL-TBB group required hospitalization. One had grade 3 anorexia, and the other had grade 3 pneumonia. In contrast, three patients in the non-CL-TBB group were hospitalized. Two had grade 3 hypoxemia, and one had grade 3 pneumonia. No pneumothorax, bleeding, or bronchial tears that needed therapeutic intervention occurred in either group. These findings suggest that CL-TBB is a safe and feasible technique for patients with peripheral pulmonary lesions.

## Discussion

The recent widespread use of CT scans and other imaging technologies has increased the number of patients diagnosed with small peripheral pulmonary lesions. This trend has resulted in a compelling need for bronchoscopists to collect an appropriate volume of samples for accurate pathological diagnosis of these lesions. Several new bronchoscopic techniques have been developed recently to address this need, resulting in a substantially increased diagnostic yield of peripheral pulmonary lesions. For example, since the introduction of RP-EBUS-guide sheath, virtual bronchial navigation, ultrathin bronchoscope, robot bronchoscope, and cone-beam CT, the diagnostic yield of peripheral pulmonary lesions has increased by 60–80%^1–3,11–13^. However, there is still room for improvement. For instance, a biopsy performed with an RP-EBUS image within the lesion means that a tissue sample was collected from inside the lesion, and thus, theoretically, the pathology should be diagnosed in all cases. However, in some cases, the pathology of the lesion is not diagnosed due to insufficient tissue volume, uneven distribution of pathological areas, or poor technical approach^14^. The thin bronchoscope with RP-EBUS-guide sheath is most commonly used to approach peripheral lung lesions and collect tissue samples by transbronchial biopsy using a 1.5-mm forceps. The main problem with this technique is that the 1.5-mm forceps can only collect a small volume of tissue^7^. The standard 1.9-mm forceps can also be used with the thin bronchoscope but without the guide sheath. However, a previous prospective study has shown that the diagnostic yield is better using a guide sheath, which only allows using a 1.5-mm forceps^3^. The guide sheath enables highly reproducible transbronchial biopsy of peripheral lung lesions. Another prospective study has demonstrated that patients undergoing bronchoscopy with the ultrathin bronchoscope had significantly higher accessibility to peripheral lung lesions and improved diagnostic yield than those undergoing bronchoscopy with the thin bronchoscope^12^. However, the large 1.9-mm forceps cannot be used with the ultrathin bronchoscope. Therefore, techniques are urgently needed to allow reproducible transbronchial biopsy using the thin or ultrathin bronchoscope or a combination of both bronchoscopes.

Genetic analysis using NGS is becoming necessary for expanding therapeutic options in patients with NSCLC^15^. Therefore, collecting an adequate volume of high-quality tissue samples during the transbronchial biopsy is essential. Many reports have shown that NGS analysis failed due to insufficient amount or poor quality of tissues collected by transbronchial biopsy using small forceps^7^. Murakami et al. performed NGS analysis on 69 of 184 patients diagnosed with NSCLC and found that the tissue surface area and the success rate of DNA (RNA) sequencing were significantly higher when samples were collected using large forceps^7^. Thick bronchoscopes used for therapeutic purposes are inappropriate for transbronchial biopsy of peripheral lung lesions due to the small diameter of peripheral bronchial branches. We generally use a thin bronchoscope with an RP-EBUS-guide sheath in routine bronchoscopic studies^14^. When we confirm that the RP-EBUS image is within or adjacent to the lesion, we advance the bronchoscope tip as close to the lesion as possible before performing transbronchial biopsy because this increases the diagnostic yield^16^. In cases where the RP-EBUS image is within the lesion, but CL-TBB is impossible with the thin bronchoscope, we perform transbronchial biopsy through the guide sheath using 1.5-mm forceps. However, in cases where the RP-EBUS image is adjacent to the lesion or invisible, and CL-TBB is possible, we remove the thin bronchoscope and introduce the ultrathin bronchoscope to collect tissue samples by the CL-TBB technique. When the transbronchial biopsy is performed through the guide sheath, tissue samples can only be collected with forceps in one direction. As a result, the biopsy samples may contain excessive blood components with less lesional tissue because they are only from one portion of the lesion. However, when CL-TBB is performed after removing the guide sheath and inserting a 1.9-mm forceps, tissue samples can be collected from different portions of the lesion by changing the angle of the bronchoscope tip.

A prospective study has shown that using a thin bronchoscope with a guide sheath under RP-EBUS guidance improves the histopathological diagnostic yield^3^. Therefore, the 1.5-mm forceps should be replaced by standard (1.9-mm) forceps only when the bronchoscope tip can be confirmed with the RP-EBUS image. Moreover, in cases where the RP-EBUS is adjacent to the lesion, and the CL-TBB technique is possible, the RP-EBUS image can be changed to within the lesion by adjusting the angle of the bronchoscope tip. The CL-TBB technique improved the histopathological diagnostic yield among eligible patients in the present study. Furthermore, in 94 NSCLC cases where CL-TBB was possible, the suitability of the specimens for NGS analysis was significantly increased, and the overall and histopathological diagnostic yields were high compared to controls.

This study showed that the CL-TBB technique, the RP-EBUS image within the lesion, the use of 1.9-mm forceps, and the number of samples with malignant cells were determinant factors for the suitability of collected specimens for NGS analysis. According to logistic regression analysis, the main predictors of suitable NGS specimens were the CL-TBB technique and the number of samples with cancerous tissues. Large lesions and RP-EBUS images within the lesion are generally known to improve the diagnostic yield of peripheral pulmonary lesions. However, in this study, the NGS suitability rate of collected specimens did not depend on the lesion size. Notably, there were cases where the bronchoscope lens directly touched the lesions, and the lesions could not be directly observed due to clot formation.

Complications previously reported during bronchoscopy include bleeding in the airways, pneumothorax, and pneumonia^3,6,17^. Intrabronchial hemorrhage may occur after a biopsy. Five samples are usually collected for diagnosis of peripheral pulmonary lesions. In this study, we collected 10 biopsy samples and observed no increased complications compared to previous studies. After tissue sampling, we kept the bronchoscope tip, and the guide sheath wedged at the biopsy site for 3 min to prevent local bleeding after the biopsy. The current bronchoscopic procedures are safer than previous conventional procedures because selective wedge of the peripheral bronchial branch is much easier with small-diameter bronchoscopes, and compressive hemostasis can be done using the guide sheath.

The limitations of this study are its retrospective nature, single-institution setting, and small sample size. Therefore, future studies with larger populations are needed to confirm these findings.

## Conclusion

This study demonstrates that the CL-TBB significantly improves the suitability of specimens collected by bronchoscopy for NGS analysis.

### Supplementary Information

Supplementary Legends.

Supplementary Video 1.

## Data Availability

All data obtained during the current study are available from the corresponding author upon reasonable request.

## Abbreviations

CL-TBB: Close-to-lesion transbronchial biopsy
NSCLC: Non-small cell lung cancer
RP-EBUS: Radial probe endobronchial ultrasound
NGS: Next generation sequencing
CT: Computed tomography
