# Supplementary material for: Close-to-lesion transbronchial biopsy: a novel technique to improve suitability of specimens for genetic testing in patients with peripheral pulmonary lesions
Source: Sci Rep. 2023 Sep 7;13:14724. doi: 10.1038/s41598-023-41726-w (PMC10485017; doi:10.1038/s41598-023-41726-w)
Supplement: Supplementary file 1 — Supplementary Legends. [file 41598_2023_41726_MOESM1_ESM.docx]

**Legend for video: Close-to-lesion transbronchial biopsy using a thin bronchoscope.** This video shows the biopsy procedure for the patient with a right middle lobe nodule in Figure 3. A thin bronchoscope was inserted into the bronchial segment and advanced as close as possible to the target lesion under radial-probe endobronchial ultrasound (RP-EBUS) and fluoroscopy guidance. Initially, the RP-EBUS image was adjacent to the lesion despite several attempts to move the probe within the lesion. However, by advancing the bronchoscope tip closer to the lesion using the guide sheath as a rail and moving it in different directions, the RP-EBUS image changed from adjacent to within the lesion. Then, the RP-EBUS was removed, and a 1.5-forceps was inserted through the guide sheath to collect five tissue samples. The guide sheath was then removed, and a standard 1.9-mm forceps was inserted to collect five more tissue samples.
